# SOCIAL DISPARITIES IN INFLAMMATORY BIOMARKERS MEDIATED BY POOR SLEEP QUALITY

**DOI:** 10.1093/geroni/igac059.1438

**Published:** 2022-12-20

**Authors:** Joseph Obiagwu, Christina Mu, Soomi Lee

**Affiliations:** University of South Florida, Tampa, Florida, United States; University of South Florida, Tampa, Florida, United States; University of South Florida, Tampa, Florida, United States

## Abstract

This study investigated whether sleep quality mediates the relationship between race/SES and biomarkers (CRP, IL6, IL10, TNF-α). Participants in the Midlife in the United States Study (n=1,689; Mage=53.02) completed the Pittsburgh Sleep Quality Index and provided information on eight life-course indicators to measure SES. Black individuals and those with lower SES had poorer sleep quality and higher inflammation compared to their counterparts. Poorer sleep quality mediated the relationship between being Black and higher CRP (β=0.02, 95%CI [0.002, 0.04]), IL6 (β=0.008, 95%CI [0.0002, 0.02]), IL10 (β=0.008, 95%CI [0.0004, 0.02]), and TNF-α (β=0.004, 95%CI [0.0002, 0.01]). Poorer sleep quality also mediated the relationship between lower SES and higher CRP (β=-0.01, 95%CI [-0.01, -0.001]), IL6 (β=-0.003, 95%CI [-0.007, -0.00]), IL10 (β=-0.003, 95%CI [-0.01, - 0.0003]), and TNF-α (β=-0.002, 95%CI [-0.004, -0.0002]). Improving sleep quality may help reduce the risk of inflammation in at-risk groups and subsequently reduce health disparities.

